# The relationship between motor skills and social development in 6–12 years old typically developing children: a systematic review

**DOI:** 10.3389/fpsyg.2026.1845725

**Published:** 2026-08-17

**Authors:** Renke He, Yulan Zhou, Shuyang Chen, Xuefen Dong, Xiandan Ye, Chi Xue

**Affiliations:** College of Physical Education and Health Sciences, Zhejiang Normal University, Jinhua, Zhejiang, China

**Keywords:** fine motor skills, gross motor skills, social behavior, social cognition, social emotion, systematic review

## Abstract

**Background:**

Motor skill development plays a pivotal role in children’s holistic development, including both physical fitness and social competence. This systematic review examined the specific relationships between motor skill components (gross and fine motor skills) and three core domains of social development (social cognition, social emotion, and social behavior) in typically developing children aged 6–12 years.

**Methods:**

We conducted a systematic search across eight databases (Web of Science, Scopus, EBSCOhost, PubMed, ScienceDirect, CNKI, Wanfang, and VIP) from inception through April 2026. After initial title/abstract screening, full texts were reviewed against inclusion criteria. Two researchers independently performed study selection and quality assessment, with a third researcher resolving discrepancies.

**Results:**

From 8,252 initially identified records, 22 studies met inclusion criteria. Analysis revealed: (1) overall motor skills showed weak-to-moderate positive correlations (r = 0.008–0.549) with social development; (2) gross motor skills demonstrated stronger associations (weak-to-strong; r = 0.198–0.762) than fine motor skills (weak-to-moderate; r = 0.060–0.534); (3) research focus was unevenly distributed, with self-perception being most studied (59.1%), followed by peer relationships, problematic behaviors, and negative emotions (18.2% each), adaptive behaviors (13.6%), and emotion recognition (4.5%).

**Conclusion:**

Findings suggest that motor skills are consistently associated with social development outcomes, with particularly strong evidence for gross motor skills. Future research should investigate underlying mechanisms and long-term effects through longitudinal designs.

**Systematic review registration:**

The complete registration record is available at: https://www.crd.york.ac.uk/PROSPERO/view/CRD42025645601. This systematic review was prospectively registered in PROSPERO on February, 2025 under the registration number CRD42025645601.

## Introduction

Social development, a multifaceted and dynamic process, is a fundamental aspect of human ontogeny that begins in early childhood and continues throughout the lifespan (Parke and Clarke-Stewart, 2010; Kostelnik et al., 2009). This developmental process involves the progressive acquisition and refinement of an individual’s ability to engage in meaningful social interactions, understand complex social norms, and adapt to diverse social environments. Contemporary developmental psychology identifies three interrelated dimensions of social development: (1) social cognition (the ability to interpret mental states in oneself and others), (2) social behavior (the manifestation of interpersonal skills, including prosocial engagement and conflict resolution), and (3) social emotion (the capacity to regulate emotions in response to situational demands) (Rantanen et al., 2012; Jalilinasab et al., 2022).

The development of these social competencies is a critical determinant of childhood well-being and long-term outcomes. Strong social development fosters meaningful relationships, academic success, and psychological resilience (John, 2001). Children with advanced social skills demonstrate better collaborative problem-solving, adaptability in novel social situations, and effective emotional regulation (Lafavor, 2018). Conversely, deficits in social functioning increase the risk of adverse outcomes, including social marginalization, peer victimization, and maladaptive behaviors (Campbell et al., 2012; Scarpa et al., 2012). Such impairments are also linked to heightened vulnerability to psychological disorders, particularly depression and anxiety, as well as aggressive and antisocial tendencies (Bandura, 1997; Rubin et al., 2006). Recent studies report concerning trends, with rising rates of developmental delays characterized by immature social awareness, poor interaction skills, and reduced resilience (Gerow et al., 2024; de Araújo, 2025). These findings highlight the need to identify modifiable factors that can enhance childhood social development.

Motor skills, the ability to execute coordinated movements through the integrated function of the nervous and musculoskeletal systems, have emerged as a key factor in children’s social development (Adolph and Hoch, 2019; Flores et al., 2023; Burton et al., 2023). As a fundamental component of behavior and development, motor skill facilitates social engagement and interpersonal interaction (Ourda et al., 2025). Proficient motor skills support the acquisition of essential social competencies (Macdonald et al., 2016). Extensive research has explored the relationship between motor skills and social development, with early studies focusing primarily on children with developmental coordination disorder (DCD). Cross-sectional evidence indicates that DCD correlates with socioemotional difficulties, including anxiety and depression (Piek et al., 2007; Skinner and Piek, 2001). Children with poor motor coordination, especially those at risk for DCD, often exhibit lower self-concept and diminished self-worth (Brown and Cairney, 2020). Additionally, fine motor impairments in DCD are strongly associated with internalizing problems (Cairney et al., 2010).

Emerging research suggests that motor skills influence social development beyond DCD, with typically developing children also benefiting from motor skill interventions (Piek et al., 2015). Recent studies have found that gross motor skills predict social–emotional outcomes, including anxiety and depression (Piek et al., 2010; Wang et al., 2022). Rigoli et al. (2012) identified a significant link between overall motor skills and emotional functioning in adolescents. Similarly, preschool children with poor fine motor skills display more hyperactive and inattentive behaviors (Pagani and Sylvie, 2012). Longitudinal studies further reveal that weaker motor skills correlate with lower social cognition (e.g., self-esteem) (Poole et al., 2018), while fine motor deficits are associated with aggressive behaviors and peer victimization (Øksendal et al., 2022).

Middle childhood represents a critical period for both motor skill acquisition and social development, with physical education (PE) serving as a primary context in which these intertwined processes unfold (Bailey, 2006; Kirk, 2010). Within PE lessons, children participate in structured movement tasks that simultaneously require physical competence and interpersonal coordination, thereby creating distinctive opportunities for fostering growth across both domains (Rovegno and Dolly, 2006). Beyond formal PE classes, school-based physical activities, including recess periods and organized sports, offer naturalistic settings where gross motor skills such as running, throwing, and catching are practiced within socially interactive environments, potentially magnifying the developmental benefits of motor competence (Lubans et al., 2010). Moreover, PE contexts are inherently social arenas in which youngsters navigate peer relationships, confront successes and setbacks publicly, and construct self-perceptions through comparative evaluations with classmates (Tietjens et al., 2020). Given these dynamics, a thorough understanding of motor–social development linkages is highly relevance for PE pedagogy and curriculum design, as such insights can guide teachers in designing activities that concurrently advance physical proficiency and social–emotional learning objectives (Dyson and Casey, 2023).

Despite growing evidence linking motor skills to social development, the precise nature of this relationship remains unclear. Most studies examine only one or two motor skill types (e.g., gross or fine motor skills) or focus on overall motor skills rather than multidimensional associations (Wang et al., 2022; Burton et al., 2023). Additionally, existing research has largely concentrated on either atypically developing children (e.g., those with DCD or autism spectrum disorder [ASD]) or specific age groups, such as preschoolers (Li et al., 2026) or adolescents (Burton et al., 2023). For example, Burton et al. systematically reviewed motor skills’ impact on social–emotional functioning in adolescents (11–18 years) (Burton et al., 2023), while Wang et al. (2022) explored gross motor skills and social skills in children with ASD. However, no systematic review has specifically investigated the relationship between motor skills and social development in children aged 6–12 years. This age range represents a critical developmental period for refining self-assessment and social interaction abilities (Lalonde and Chandler, 1995), and it is also the phase when PE instruction becomes more structured and skill-oriented. Given the substantial physiological and psychological differences between childhood and adolescence (Lloyd et al., 2015), findings from other age groups may not generalize to this population. A focused examination of 6-12-year-olds is essential to inform targeted interventions that enhance both motor and social development, particularly in educational settings where PE teachers can directly influence motor and social outcomes through curriculum design and instructional practices.

Given embodied cognition theory (Adolph and Hoch, 2019) and prior evidence, specific predictions emerge. First, overall motor skills show weak-to-moderate positive links with social-cognitive, behavioral, and emotional outcomes (Leonard and Hill, 2014; Mancini et al., 2019). Second, gross motor skills (e.g., running) are more directly tied to peer interaction than fine motor skills (e.g., handwriting), so they should exhibit stronger associations. Third, self-perception, most proximal to motor-based mastery (Bandura, 1997), should show the strongest link, while emotion recognition, mediated by indirect pathways (Cummins et al., 2005), the weakest. Accordingly, we hypothesize:

*H1*: Overall motor skills are positively associated with social cognition, behavior, and emotion (weak-to-moderate effects).

*H2*: Gross motor skills show stronger associations than fine motor skills.

*H3*: Motor skills associate most strongly with self-perception and least with emotion recognition.

## Methods

### Search strategy

This systematic review strictly adhered to the Preferred Reporting Items for Systematic Reviews and Meta-Analyses (PRISMA) guidelines (Page et al., 2022). The study protocol was prospectively registered on PROSPERO (registration number: CRD42025645601) prior to commencing the review. The completed PRISMA checklist, including page numbers for relevant sections, is provided in Supplementary Table 1. A comprehensive systematic search was conducted across eight international databases (Web of Science, Scopus, EBSCOhost, PubMed, ScienceDirect, CNKI, Wanfang, and VIP), covering all available records from their inception until April 2026. The search strategy employed four keyword clusters: (1) motor skills (motor skill OR motor competenc* OR motor development OR gross motor* OR fine motor* OR object control OR locomotor* OR stabil* skill* OR motor coordinat*), (2) social development (social development OR social competenc* OR social skills OR self-concept OR self-regulation OR self-perception OR emotion* OR depressi* OR anxiety* OR prosocial behavio* OR aggressive behavio* OR social withdrawal), (3) population (elementary school OR primary school OR middle school OR Youth OR Teen* OR Student* OR Child*), and (4) study focus (correlat* OR determinant* OR predictor* OR relationship* OR association* OR influence OR factor*). To ensure the identification of all relevant studies, bibliographic screening and forward citation searching of previous reviews and included studies were performed to capture articles that might not have been retrieved by the initial search criteria.

### Study selection

Following removal of duplicate studies, two researchers (RH and SC) independently screened titles and abstracts against predefined eligibility criteria. Publications meeting ≥1 exclusion criterion at this stage were systematically excluded, while potentially eligible studies progressed to full-text review. Researchers independently evaluated all retained full-text articles using the standardized inclusion/exclusion framework. Inter-rater reliability was excellent (Cohen’s kappa coefficient *κ* = 0.86). A senior researcher arbitrated any unresolved discrepancies. This study adhered to the PICOS criteria: P (Population) – Typically developing children aged 6–12 years; I (Exposure) - motor skill competence assessment; C (Comparator) - varying levels of motor skill; O (Outcomes) – standardized measures of social development; and S (Study design) – observational (cross-sectional/longitudinal) studies in Chinese or English.

Studies were selected based on the following criteria: (1) studies investigating the relationship between motor skills and at least one dimension of social development were included, while those not addressing this theme were excluded; (2) studies reporting quantitative data (e.g., correlation coefficients) on the association between motor skills and social development were included, whereas those lacking detailed numerical evidence were excluded; (3) observational studies (cross-sectional or longitudinal designs) were included, while intervention studies, reviews, qualitative research, and case reports were excluded; (4) studies focusing on typically developing children aged 6–12 years (primary school age) were included, whereas those examining other age groups (e.g., preschool, adolescents) or clinical populations (e.g., individuals with developmental disorders or mental health conditions) were excluded; (5) peer-reviewed articles published in Chinese or English were included, while non-peer-reviewed works, unpublished data, conference abstracts, dissertations, and studies without full-text availability were excluded.

### Quality assessment

The methodological quality of included studies was assessed using the McMaster Critical Review Form for Quantitative Studies (Law et al., 1998), consistent with established practices (Bloemen et al., 2015; Mortimer et al., 2014). This 14-item tool evaluates key study components including research objectives, background literature, and study design. Each item was scored dichotomously (“Yes” = 1 point; “No” = 0 points), yielding total scores ranging from 0 to 14. Based on these scores, studies were classified into three quality tiers: low (0–6 points), moderate (7–10 points), and high (11–14 points) (Katrak et al., 2004; Burger and Louw, 2009). To maximize objectivity, we implemented a dual-independent blinded assessment protocol. Two researchers (XD and XY) independently applied the appraisal framework, with inter-rater reliability measured using *κ* against predetermined concordance thresholds. A senior researcher resolved any discrepancies through consensus review.

### Data extraction and synthesis

The lead author (RH) performed initial data extraction using a standardized Microsoft Excel spreadsheet specifically designed for this study. The extracted data encompassed first author’s name, publication year, country, research design, sample characteristics (size, gender distribution, and age range), follow-up duration for longitudinal studies, measurement methods for motor skills and social development, confounding factors, and key findings. Given substantial methodological heterogeneity and variability in assessment tools across studies, we determined that conventional meta-analysis would be inappropriate. Instead, we adopted the correlation coefficient (r) as our common effect size metric. For studies reporting different statistical values (F, T, or *χ*^2^), we implemented established conversion formulas: [ $r=\sqrt{\frac{t^{2}}{t^{2}+\mathrm{df}}}$ ; $r=\sqrt{\frac{F}{F+{\mathrm{df}}_{e}}}$ ; $r=\sqrt{\frac{\chi^{2}}{\chi^{2}+N}}$ ; $r=\beta +0.05\left(\beta \geq 0\right)$ ; $\;r=\beta \left(\beta <0\right)$ ( $\beta \in \left(-0.5,0.5\right)$ )]. When correlation matrices were reported, we computed synthesized coefficients using $r_{\mathrm{xy}}=\frac{\sum r_{x_{i}}r_{y_{j}}}{\sqrt{n\left(n-1\right)\bar{r_{x_{i}x_{j}}}}\sqrt{m+\left(m-1\right)\bar{r_{y_{i}y_{j}}}}}$ . This standardized approach ensured consistent effect size estimation across studies despite variations in methodologies and reporting formats. The strength of associations between motor skills and social development outcomes was interpreted according to established criteria by Iversen and Gergen (1997), where correlation coefficients (r) ≥ 0.75 indicated strong relationships, values between 0.30 and 0.75 represented moderate correlations, and coefficients ≤ 0.25 were considered weak.

## Results

### Included studies

**Figure 1:**
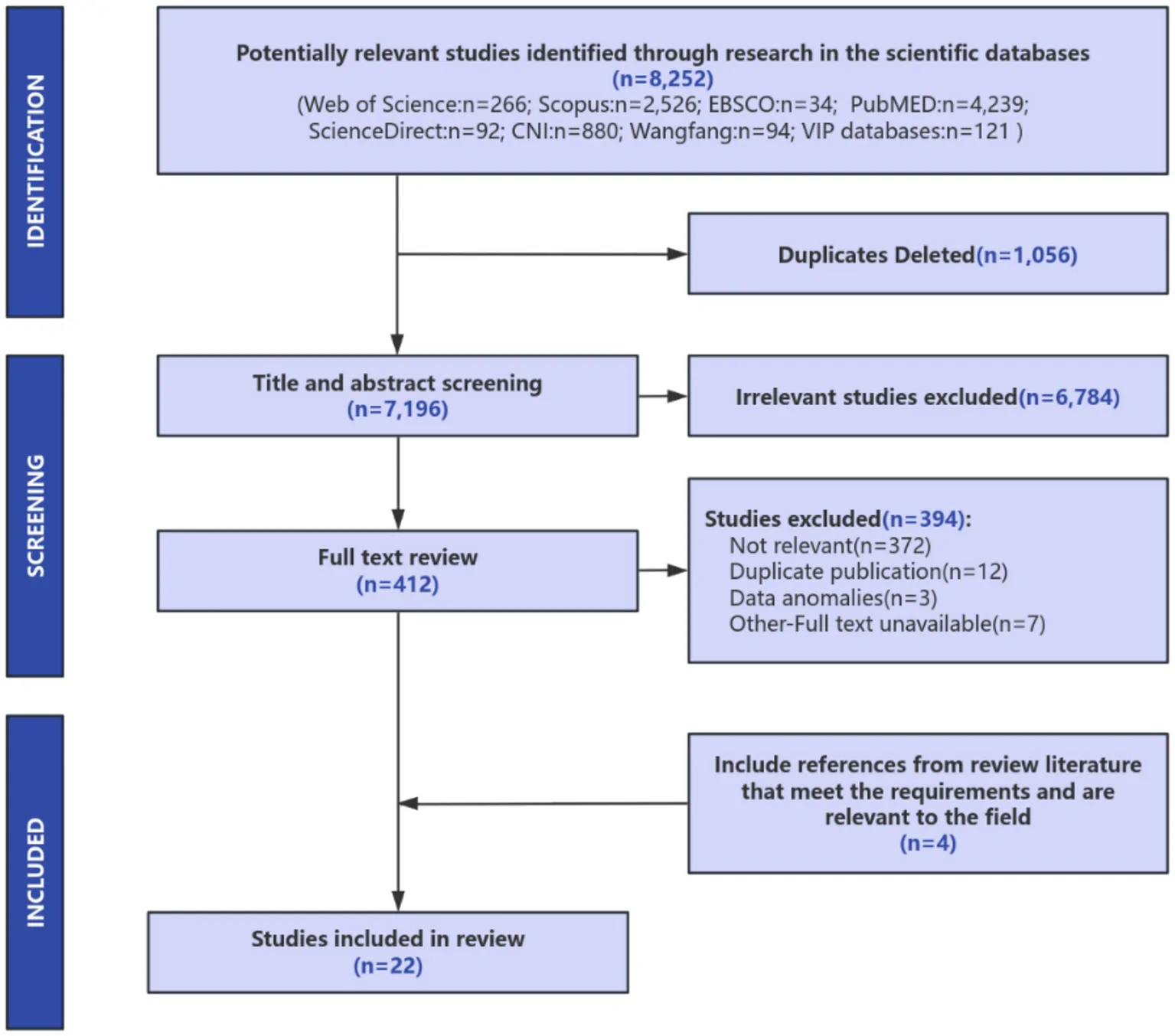
PRISMA flow diagram of the systematic review process.

Our systematic search across eight databases initially identified 8,252 records (Web of Science: 266; Scopus: 2,526; EBSCO: 34; PubMed: 4,239; ScienceDirect: 92; CNKI: 880; Wanfang: 94; VIP: 121). After removing 1,056 duplicates using automated deduplication algorithms, we screened the remaining 7,196 records by title and abstract, excluding 6,784 studies that did not meet our criteria. This left 412 articles for full-text evaluation. Through rigorous assessment, we excluded 394 publications that failed to satisfy our inclusion criteria, resulting in 18 eligible studies. We identified four additional relevant studies through bibliography screening and forward citation searching. The final analysis included 22 publications. Figure 1 presents the complete PRISMA-compliant selection workflow, detailing the attrition metrics at each screening stage.

### Methodological quality

Inter-rater reliability for the double independent blind assessments showed strong agreement, with kappa coefficients (*κ*) ranging from 0.438 to 1.000 (M = 0.766), reflecting moderate to perfect concordance. All included studies demonstrated foundational scientific integrity, including explicitly defined research objectives, comprehensive integration with existing literature, complete reporting of statistical significance values (*p*-values), appropriate application of analytical methods, and conclusions that followed logically from the presented evidence. Key quality indicators revealed that 95.5% (*n* = 21) employed robust experimental designs, with 90.9% (*n* = 20) providing complete sample characterizations and 86.4% (*n* = 19) demonstrating ethical compliance through documented informed consent procedures. Psychometric validation was confirmed for instruments used in 81.8% (*n* = 18) of studies, while 77.3% (*n* = 17) offered evidence-based recommendations extending beyond their immediate findings. Notably, 59.1% (*n* = 13) addressed both practical implications and methodological limitations, though only 9.09% (*n* = 2) conducted statistical power analyses to verify sample adequacy. When stratified by methodological rigor, 77.3% (*n* = 17) met high-quality standards (11–14 points), with the remaining 22.7% (*n* = 5) classified as moderate quality (7–10 points) (Supplementary Table 2).

### Studies characteristics

The characteristics of the 22 included studies are presented in Supplementary Table 3. Geographically, most studies originated from English-speaking countries (77.3%, *n* = 17), whereas only 22.7% (*n* = 5) come from Chinese-speaking regions. The publication dates ranged from 1997 to 2025, with more than half (*n* = 12) concentrated in the 2011–2020 decade. The remaining studies included earlier (27.3%, *n* = 6, pre-2011) and more recent (18.2%, *n* = 4, post-2020) publications. Sample sizes showed considerable variation across studies, ranging from 50 to 23,215. Among the 20 studies (90.9%) that reported gender composition, samples were distributed as follows: gender-balanced cohorts (45.0%, *n* = 9), female-dominant groups (30.0%, *n* = 6), and male-predominant samples (25.0%, *n* = 5). Methodologically, cross-sectional designs predominated (68.2%, *n* = 15), while 31.8% (*n* = 7) employed longitudinal approaches with varying observation periods: short-term (42.9%, *n* = 3, <5 years), medium-term (42.9%, *n* = 3, 5–10 years), and long-term (14.2%, *n* = 1, ~30 years). Multivariate analyses included comprehensive covariate adjustments for demographic/biological parameters (gender, age, ethnicity, anthropometrics, BMI), psycho-cognitive domains (attentional capacity, emotional regulation), behavioral competencies (academic performance), and sociocultural determinants (familial socio-economic status [SES]). Covariate adjustment was most prevalent for gender (81.8%, *n* = 18), age (59.1%, *n* = 13), and familial SES (27.8%, *n* = 5).

To contextualize the evidence for PE, we classified included studies according to their primary setting. The vast majority of studies (95.5%, *n* = 21) assessed motor skills and social outcomes in school or educational settings through school-based assessment. In contrast, only one study (4.5%) employed a community-based or clinical-adjacent sample (Bejerot and Humble, 2013). This distribution indicates that most evidence derives from school contexts, supporting its relevance to PE and educational practice. However, notably, only one study (Livesey et al., 2011) explicitly assessed outcomes during PE lessons; the remainder conducted general school-based assessments without specifying the PE context.

### Associations between motor skills and social development

Table 1 presents a synthesis of methodological approaches and correlational strengths from 22 studies examining motor-social development relationships. The studies fell into three categories: (1) eight studies investigating overall motor skills in relation to social cognition, social emotion, and social behavior (Poole et al., 2018; Mancini et al., 2018; Bejerot et al., 2013; Vedul-Kjelsås et al., 2011; Livesey et al., 2011; Duncan et al., 2018; Diao et al., 2017; Yuan et al., 2019); (2) eleven studies analyzing gross motor skills and social development (Piek et al., 2010; Øksendal et al., 2022; Chen et al., 2024; Liu et al., 2021; Tietjens et al., 2020; Miklankova, 2017; Crane et al., 2017; Livesey et al., 2011; Ommundsen et al., 2010; Piek et al., 2006; Zhang et al., 2025); and (3) eight studies exploring fine motor skills and social development (Brown and Cairney, 2020; Piek et al., 2010; Øksendal et al., 2022; Lee et al., 2020; Livesey et al., 2011; Piek et al., 2006; Cummins et al., 2005; Rose et al., 1997).

**Table 1:** Summary of studies examining the relationship between motor skills and social development.

Author and year	Motor skills			Social development		
	Overall motor skills	Gross motor skills	Fine motor skills	Social cognition	Social behavior	Social emotion
Chen et al. (2024)		×		+		
Liu et al. (2021)		×		+		
Øksendal et al. (2022)		×	×		+	
Tietjens et al. (2020)		×		+		
Lee et al. (2020)			×		+	+
Brown and Cairney (2020)			×	0		
Poole et al. (2018)	×			+		
Miklankova (2017)		×			0	
Crane et al. (2017)		×		+		
Mancini et al. (2018)	×					+
Bejerot et al. (2013)	×				+	
Vedul-Kjelsås et al. (2011)	×			+		
Livesey et al. (2011)	×	×	×	+		
Piek et al. (2010)		×	×			+ Gross 0 Fine
Ommundsen et al. (2010)		×		+		
Piek et al. (2006)		×	×	+		
Cummins et al. (2005)			×		+	+
Rose et al. (1997)			×	+		
Diao et al. (2017)	×			+		
Duncan et al. (2018)	×			+		
Yuan et al. (2019)	×			+		
Zhang et al. (2025)		×			+	
Total	5	11	8	14	6	4

×, the specific dimensions of motor skills mentioned in the main conclusions of the literature; +, positive correlation; 0, not related.

The distribution of reported correlation coefficients was systematically examined. For overall motor skills, coefficients ranged from r = 0.008 to 0.549 (median: 0.29), with most studies reporting weak-to-moderate positive correlations. For gross motor skills, coefficients showed wider variability, ranging from r = 0.198 to 0.762 (median: 0.41), indicating consistently stronger and more diverse associations with social development outcomes. For fine motor skills, coefficients ranged from r = 0.060 to 0.534 (median: 0.24), reflecting more modest and relatively consistent positive associations. Among gross motor skill studies, 91% (10/11) demonstrated weak-to-strong positive correlations, while 75% (6/8) of fine motor skill studies showed weak-to-moderate positive associations. When examining social development dimensions, the strongest correlations were observed between motor skills and self-perception (r range: 0.21–0.76), whereas weaker correlations were found for negative emotions (r range: 0.01–0.41) and adaptive behaviors (r range: 0.20–0.40). This variation in effect sizes across motor skill types and social development domains underscores the importance of differentiated analysis and suggests that the strength of associations depends on how both motor and social constructs are operationalized and measured.

**Figure 2:**
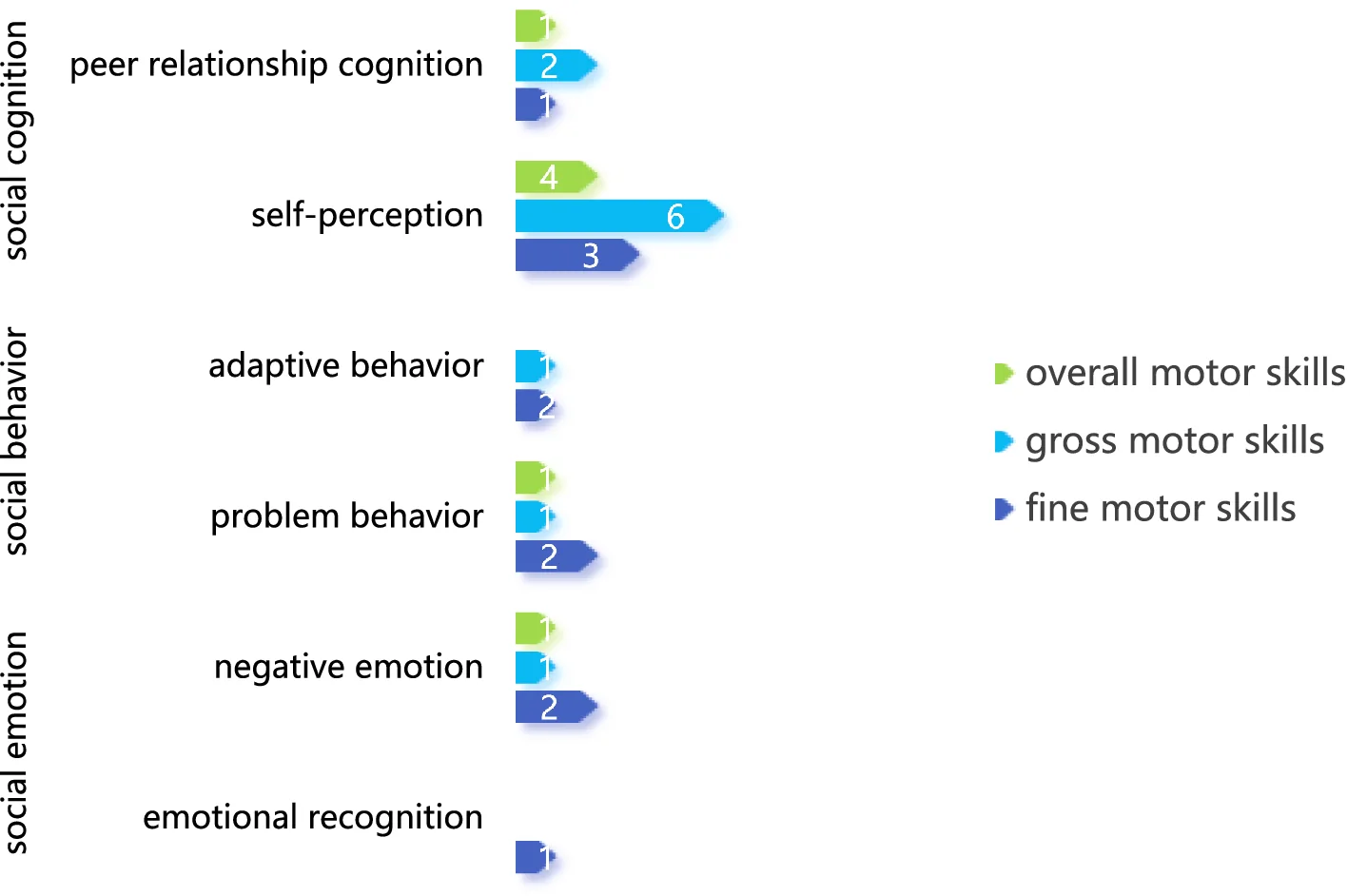
Distribution of studies associating specific motor skill components with social development dimensions.

Figure 2 illustrates relationships between specific motor skill components and social development dimensions. Studies revealed distinct patterns: overall motor skills primarily related to peer relationship cognition, self-perception, problematic behaviors, and negative emotions; gross motor skills showed broader associations including these plus adaptive behaviors; while fine motor skills connected with all these domains plus emotion recognition. Self-perception emerged as the most extensively investigated social development indicator, examined in 59.1% of studies (*n* = 13). Peer relationship cognition, problematic behaviors, and negative emotions were each studied in 18.2% of cases (*n* = 4 for each domain). Less frequently addressed were adaptive behaviors (13.6%, *n* = 3) and emotion recognition, which was analyzed in only one study (4.5%).

### Assessment tools for motor skill and social development

Table 2 summarizes the assessment tools used across studies, with nine instruments measuring motor skills and eleven evaluating social development. For motor skills, the most frequently used tools included the Movement Assessment Battery for Children (MABC; 22.7%, 5 studies) and Test of Gross Motor Development (TGMD; 40.1%, 9 studies), followed by the McCarron Assessment of Neuromuscular Development (MAND; 13.6%, 3 studies). Overall motor skills were primarily assessed using the Bruininks–Oseretsky Test of Motor Proficiency-Short Form (BOTMP-SF) and MABC, while gross motor evaluations relied on TGMD versions, MABC, the Child Development Inventory (CDI), the Körper Koordinationstest für Kinder (KTK), the Ages and Stages Questionnaires (ASQ), and MAND. Fine motor skills were measured using MABC, CDI, and MAND. For social development, Harter’s Self-Perception Profile for Children (SPPC; 27.3%, 6 studies) was the predominant tool, particularly for assessing social cognition alongside the Pictorial Scale of Perceived Movement Skill Competence for Young Children (PSPC, 22.7%, 5 studies). Social behavior was evaluated using the Korean standardized version of the Behavior Assessment System for Children (KBASC), the Piers-Harris Children’s Self-Concept Scale, Social Skills Improvement System Rating Scales (SSIS), and the Child Behavior Checklist (CBCL), while social emotion measures included Emotion Recognition Scales (ERS), the Strengths and Difficulties Questionnaire (SDQ), KBASC, and CBCL. The distribution of tools reflected methodological diversity, with SPPC emerging as the most common social measure and MABC/TGMD as the primary motor assessments.

**Table 2:** Assessment tools for evaluating motor skills and social development in included studies.

Author and year	Motor skills		Social development	
	Instrument	Evaluation	Instrument	Evaluation
Chen et al. (2024)	TGMD-3	GMS	SPPC	Social cognition (self-perception)
Liu et al. (2021)	TGMD-3	GMS	PSPC	Social cognition (self-perception)
Øksendal et al. (2022)	CDI	GMS, FMS	Questionnaire	Social behavior (problem behavior)
Tietjens et al. (2020)	TGMD-3	GMS	PSPC	Social cognition (self-perception)
Lee et al. (2020)	MABC-2	FMS	KBASC-2	Social emotion (negative emotion), Social behavior (adaptive behavior)
Brown and Cairney (2020)	BOTMP-SF	FMS	SPPC	Social cognition (self-perception)
Poole et al. (2018)	BOTMP-SF	MS	GSW (SPPC)	Social cognition (self-perception)
Miklankova (2017)	TGMD-2	GMS	Piers-Harris Children’s Self-Concept Scale	Social behavior (adaptive behavior)
Crane et al. (2017)	TGMD-2	GMS	PSPC	Social cognition (self-perception)
Mancini et al. (2018)	MABC-2	MS	SDQ-P	Social emotion (negative emotion)
Bejerot et al. (2013)	Questionnaire	MS	Questionnaire	Social cognition (self-perception)
Vedul-Kjelsås et al. (2011)	MABC	MS	SPPC	Social cognition (self-perception)
Livesey et al. (2011)	MABC	MS, GMS, FMS	peer rating	Social cognition (peer relationship cognition)
Piek et al. (2010)	MABC、ASQ	GMS, FMS	CBCL	Social emotion (negative emotion)
Ommundsen et al. (2010)	KTK	GMS	Nomination process	Social cognition (peer relationship cognition)
Piek et al. (2006)	MAND	GMS, FMS	SPPC	Social cognition (self-perception)
Cummins et al. (2005)	MAND	FMS	ERS, CBCL	Social emotion (emotional recognition), social behavior (problem behavior)
Rose et al. (1997)	MAND	FMS	SPPC	Social cognition (self-perception)
Diao et al. (2017)	TGMD-3	MS	PMSC	Social cognition (self-perception)
Duncan et al. (2018)	TGMD-2	MS	PSPC	Social cognition (self-perception)
Yuan et al. (2019)	TGMD-3	GMS	PSPC	Social cognition (self-perception)
Zhang et al. (2025)	TGMD-3	GMS	SSIS	Social behavior

MS, overall motor skills; GMS, gross motor skills; FMS, fine motor skills; TGMD, Test of Gross Motor Development; CDI, Child Development Inventory; MABC, the Movement Assessment Battery for Children; BOTMP-SF, The Bruininks–Oseretsky Test of Motor Proficiency-Short Form; ASQ, Ages and Stages Questionnaires; KTK, The Körper Koordinationstest für kinder, Body Coordination Test for Children; MAND, McCarron Assessment of Neuromuscular Development; SPPC, Harter’s Self-Perception Profile for Children; PSPC, The Pictorial Scale of Perceived Movement Skill Competence for Young Children; KBASC-2, the Korean standardized version of the Behavior Assessment System for Children, Second Edition; GSW, Harter Self-Perception Profile for Adolescents global self-worth subscale; SDQ-P, Strengths and difficulties questionnaire; CBCL, Child Behavior Checklist; ERS, Emotion Recognition Scales; PMSC, Pictorial Scale of Perceived Movement Skill Competence; SSIS, Social Skills Improvement System Rating Scales.

## Discussion

This systematic review examined the specific associations between motor skill components (gross and fine motor skills) and social development in typically developing children aged 6–12 years. The evidence revealed consistent positive correlations, with all studies (100%) reporting links between social development and overall motor skills, 90.9% for gross motor skills, and 75% for fine motor skills. These findings confirm our first hypothesis (H1) that overall motor skills show weak-to-moderate positive associations with all three dimensions of social development. H2 (gross motor skills show stronger associations than fine motor skills) is also supported, while H3 (motor skills associate most strongly with self-perception and least with emotion recognition) is partially supported: the strong link with self-perception is confirmed, but the prediction regarding emotion recognition cannot be tested adequately due to insufficient data (only one study). Unlike previous systematic reviews that have focused on adolescents (Burton et al., 2023; 11–18 years) or children with developmental disorders (Wang et al., 2022), the present review specifically targets typically developing children aged 6–12 years, a critical yet underexamined developmental window for both motor skill and social functioning. Thus, it addresses a significant gap in the existing literature.

The findings of this review have direct relevance to PE practice, as the majority of included studies were conducted in school settings where PE lessons provide structured opportunities for motor and social development. Within PE, the observed association between gross motor skills and social outcomes is particularly salient, because group-based movement activities inherently require cooperation, communication, and social coordination (Rovegno and Dolly, 2006). PE teachers are uniquely positioned to facilitate motor-skill development and social–emotional learning simultaneously through carefully designed activities that promote both physical competence and positive peer interaction (Bailey, 2006).

Our review suggests that low motor competence may set off a cascade of adverse social consequences within PE environments. Children exhibiting poorer movement abilities are more vulnerable to negative peer evaluations and exclusion from group-based activities, experiences that erode their perceived physical competence and intensify feelings of social marginalization (Brown and Cairney, 2020; Øksendal et al., 2022). To avoid public embarrassment or perceived failure, such children frequently withdraw from movement-based opportunities, resulting in decreased participation and a weakened sense of belonging (Scarpa et al., 2012; Skinner and Piek, 2001). This pattern of avoidance creates a self-reinforcing cycle: diminished engagement curtails skill acquisition, which in turn sustains low perceived competence, deepens social avoidance, and entrenches inequitable participation in PE. Although correlational evidence and theoretical models support these proposed pathways, causality cannot be established without further longitudinal and experimental investigations. Moreover, because this review was restricted to typically developing children, the applicability of our conclusions to populations with developmental, health, or additional educational needs remains uncertain.

The variability in correlation coefficients observed across studies warrants careful interpretation. Notably, the widest range was found for gross motor skills (r = 0.198–0.762), which may reflect the diversity of instruments used to assess this domain as well as the variety of social outcomes examined (self-perception, peer relationships, adaptive behaviors, and problem behaviors). In contrast, fine motor skills showed a narrower range (r = 0.060–0.534), possibly due to more consistent measurement approaches and a more focused set of social outcomes. The relatively wide range for overall motor skills (r = 0.008–0.549) likely represents an aggregate of these domain-specific patterns. This observed variability cannot be attributed to sampling error alone; it is likely driven by genuine differences in the strength of associations depending on the specific motor skill component, social development dimension, and assessment instrument employed. Such heterogeneity is consistent with previous systematic reviews (Leonard and Hill, 2014; Mancini et al., 2019), and it sets the stage for further examination of differential relationships.

These findings align with Leonard and Hill’s (2014) meta-analysis of 43 studies, which established that advanced motor skills predict enhanced social-cognitive functioning across both typical and atypical developmental trajectories. Furthermore, our results corroborate Mancini et al.’s (2019) systematic review, indicating that motor skill may serve as a protective factor against internalizing symptoms such as anxiety and depression—psychological factors known to influence social engagement. While the literature on direct links between motor skills and specific social behaviors remains limited, supporting evidence comes from Bejerot and Humble’s (2013) case–control study, which found that motor deficits significantly predict maladaptive social behaviors.

The analysis revealed important distinctions in how different motor skill components relate to social development. Overall motor skills demonstrated weak-to-moderate correlations with social development outcomes. When examining specific domains, gross motor skills showed stronger associations (ranging from weak-to-strong correlations) compared to fine motor skills (consistently weak-to-moderate correlations). The robust relationship between gross motor skills and social development appears mediated by multiple mechanisms. Gross motor skills, encompassing both locomotor (e.g., running, jumping) and object control skills (e.g., throwing, catching), facilitate social development through embodied learning processes that engage children in perception-action cycles within their physical and social environments (Brian et al., 2024). The mediating role of physical activity is particularly noteworthy, as children with advanced gross motor skills typically engage in more frequent and intense activity, which in turn promotes socioemotional competence by reducing negative affect, enhancing peer interaction quality, and fostering cooperative behaviors (Sollerhed et al., 2008). In contrast, fine motor skills, which involve hand-eye coordination, manual dexterity, and precision movements, showed more modest associations with social outcomes. These skills appear primarily linked to neurocognitive processes, particularly executive functioning (Pagani et al., 2010; Roebers et al., 2014), suggesting they influence self-regulatory capacities rather than broader social competencies. While proficient fine motor skills support autonomy and self-efficacy in daily activities (Cameron et al., 2016), their weaker social correlates likely reflect their more limited role in direct social interaction compared to gross motor skills.

The current findings must also be understood within the framework provided by recent DE-PASS evidence syntheses concerning determinants of children’s physical activity (PA). Khudair et al. (2025) reviewed 38 modifiable factors, concluding that interventions have generally produced only trivial-to-moderate effects on PA outcomes—a conclusion reinforced by Ling et al. (2024), who reported no consistent evidence linking modifiable determinants to self-reported PA across any setting. In a related synthesis, Kolovelonis et al. (2024) found that determinants of adolescent PA are overwhelmingly individual-level and psychological (21 out of 33 determinants), with policy-related or environmental factors receiving minimal empirical support. Collectively, these reviews advocate for systems-oriented approaches that address determinants spanning individual, interpersonal, environmental, and policy levels. Applied to our focus on motor-social development, this evidence suggests that the weak-to-moderate associations we observed may be shaped by contextual variables including school-level influences, teaching practices, peer interactions, and family support. Consequently, future interventions should adopt multi-level strategies rather than concentrating exclusively on individual-level determinants.

Regarding specific social-cognitive dimensions, current literature reveals a significant imbalance in social development research, with self-perception studies comprising 59.1% of social cognition investigations, compared to adaptive behavioral regulation (13.6%) and socio-emotional recognition (4.5%). This distribution reflects fundamental theoretical priorities in developmental science. From a developmental systems perspective, the acquisition of socially adaptive traits through cultural internalization emphasizes individual developmental pathways (Harter, 2012). The predominance of self-perception research aligns with this framework, as self-concept development forms the foundation for subsequent social-cognitive achievements. Neuroscience research further substantiates these research priorities, demonstrating that motor-cognitive integration depends on synchronized cerebellar-prefrontal circuit activation (Xia et al., 2018). Notably, motor skill enhances prefrontal cortex activation, which supports the executive metacognitive functions essential for self-referential processing. These neurobiological findings not only justify the field’s focus on self-perception but also reveal potential neural mechanisms connecting motor development with broader social-cognitive outcomes. The evidence strongly supports the first part of our third hypothesis (H3), that self-perception shows the strongest associations with motor skills. However, the second part, that emotion recognition would show the weakest associations, could not be adequately tested due to insufficient data, and therefore remains unverified.

Beyond self-perception, empirical support for adaptive behaviors and emotion recognition remains conspicuously limited. Regarding adaptive behaviors, the three available studies have consistently documented positive associations with motor competence, suggesting that children with stronger motor skills may be better equipped to navigate complex social environments effectively. With respect to emotion recognition, however, only a single investigation has explored this dimension, revealing a positive correlation between fine motor proficiency and accuracy in identifying others’ emotional expressions, a finding that aligns with predictions derived from embodied cognition theory. The pronounced scarcity of research addressing this domain is particularly concerning, given that emotion recognition serves as a foundational component of social competence. Taken together, these gaps imply that the existing evidence base may be incomplete and potentially biased toward cognitively oriented constructs (e.g., self-perception), thereby constraining the ecological validity of the available findings.

The evidence base of this review has three notable boundaries. First, although 95.5% of studies were schoo-based, data collection typically took place during general testing sessions rather than in the naturalistic settings, such as PE lessons, recess, or playgrounds, where intervention would naturally be implemented and children’s social and motor behaviors ar mosty observable. Second, the literature skews heavily toward self-perception (59.1%), while adaptive behaviors (13.6%) and emotion recognition (4.5%) receive far less attention. This limits understanding of motor-social links in PE scenarios requiring rule following and emotional decoding. Third, evidence from unstructured school contexts (recess, free play) is nearly absent, despite peer interactions being most spontaneous there and motor competence most critical for inclusion. The DE-PASS reviews (Khudair et al., 2025; Kolovelonis et al., 2024; Ling et al., 2024; Ciaccioni et al., 2026) similarly note a disproportionate focus on individual-level psychological variables at the expense of environmental and policy-level factors—a pattern echoed in our finding that self-perception dominates the motor-social literature. Thus, conclusions are most robust for self-perception but tentative for adaptive behaviors, emotion recognition, and naturalistic PE settings. Future research must prioritize these underrepresented domains to directly inform inclusive PE practice.

These gaps point to several critical directions for future work. First, the pathways linking motor skills to socioemotional outcomes remain unclear, with current evidence suggesting indirect mediation through play-based movement competence and mastery experiences (Skinner and Piek, 2001). Second, children with motor impairments frequently exhibit affect recognition difficulties (Cummins et al., 2005), indicating an underexplored connection between fundamental movement skills and social-affective processing. Third, the field lacks robust investigations of motor skills’ influence on complex adaptive behaviors in natural social environments (e.g., playgrounds, PE classes). Finally, consistent with the DE-PASS framework’s emphasis on multi-level determinants, future research should adopt systems-based approaches that simultaneously examine individual, interpersonal, environmental, and policy-level influences on motor-social development. This would help identify the most potent intervention targets and inform comprehensive strategies for promoting inclusive PE practice.

In addition to conceptual gaps, methodological issues also warrant close attention. The studies included in this review largely relied on standardized instruments, with the TGMD (versions 2 and 3) and MABC (including MABC-2) emerging as the most common motor skill measures, whereas Harter’s SPPC was the predominant tool for assessing social development. Although these instruments possess robust psychometric properties (Webster and Ulrich, 2017, for TGMD-3; Brown and Lalor, 2009, for MABC-2; Harter, 2012, for SPPC), their application in practice is constrained by specialized equipment needs and time-consuming administration procedures (Kim et al., 2015). One clear reflection of these constraints is the marked variability in assessment tools across the 22 reviewed investigations: nine distinct instruments were employed to evaluate motor skills (TGMD: 40.1%; MABC: 22.7%) and eleven to measure social development (SPPC: 27.3%). Given that different instruments may capture the same construct in divergent ways—for instance, the TGMD assesses actual motor performance via direct observation, while the PSPC gauges children’s perceived competence—these conceptually related but methodologically distinct measures cannot be used interchangeably. As a result, the broad spectrum of reported correlation coefficients (r = 0.008–0.762) may partially arise from measurement discrepancies rather than genuine differences in the strength of the motor-social development link, highlighting the urgent need for improved instrument standardization in future research.

Future research should prioritize three key directions for the assessment methodologies: (1) development of computerized motion analysis systems to enable more ecological assessment of motor skills in natural settings, particularly in school and playground environments; (2) creation of assessment tools with age-stratified normative standards to avoid floor and ceiling effects across age ranges; and (3) establishment of standardized protocols that ensure consistent measurement of both motor and social domains across studies. Addressing these methodological challenges will be essential for advancing our understanding of the complex relationships between motor skills and social development in childhood.

### Practical implications

The findings of this systematic review offer actionable insights for educators, health professionals, and policymakers working with children aged 6–12 years. For PE teachers, the moderate-to-strong links between gross motor skills and social development suggest using structured group-based gross motor activities as platforms for social–emotional learning. For early childhood and primary teachers, fine motor difficulties may indicate social–emotional vulnerability, warranting classroom accommodations and routine screening. For school psychologists, motor-based interventions can enhance self-perception and social competence, especially in children with interpersonal difficulties. For policymakers, research gaps, particularly in adaptive behaviors and emotion recognition, should guide funding toward integrated curricula and comprehensive evaluation systems.

These associations have direct implications for inclusive PE practice. Teachers should employ differentiated instruction with multiple entry points and progressive pathways; provide mastery-oriented feedback emphasizing effort over comparison; modify tasks (e.g., larger equipment, rule changes) to ensure accessibility; use cooperative learning and peer-mediated supports (e.g., buddy systems) to foster positive peer relationships; and prepare educators through training on the motor–social link and inclusive pedagogies. Establishing psychologically safe environments, by minimizing public performance evaluation, normalizing mistakes as learning, avoiding elimination games, and intervening against teasing, reduces social marginalization and supports positive social–emotional outcomes for all children.

### Limitations

Several methodological constraints warrant acknowledgement. First, the predominance of cross-sectional designs (68.2%) precludes causal inferences, while the limited longitudinal studies (31.8%) vary substantially in follow-up intervals and methodology, complicating interpretation. Second, restricting inclusion to Chinese- and English-language publications may introduce language bias and limit cultural representation. Third, marked heterogeneity in assessment instruments—nine motor skill measures and eleven social development measures—constrains cross-study comparability and precluded meta-analysis or subgroup analyses. Fourth, as discussed earlier, the disproportionate emphasis on self-perception (59.1% of studies) relative to adaptive behaviors (13.6%) and emotion recognition (4.5%) restricts the breadth of conclusions, particularly for the latter two theoretically important yet understudied domains. Fifth, notable variability across samples in size (50 to 23,215), age range (6–12 years), and cultural contexts, coupled with inconsistent control for confounders (e.g., socioeconomic status, physical activity), may attenuate generalizability. Sixth, although 95.5% of studies were school-based, most assessed motor-social relationships in general testing sessions rather than during authentic PE lessons, recess, or playground activities, reducing ecological validity for PE practice. Seventh, the evidence base is restricted to typically developing children, substantially limiting generalizability to populations with DCD, ASD, intellectual disabilities, or other neurodevelopmental conditions. Future research should examine these associations in clinical and mixed-ability populations as well as within authentic educational contexts, including PE classes and playgrounds.

## Conclusion

This systematic review provides the first comprehensive synthesis of motor-social development relationships specifically in typically developing children aged 6–12 years, offering novel evidence that differentiates the strengths of associations across motor skill components and social development dimensions. The review of 22 studies reveals three key findings: (1) overall motor skills show weak-to-moderate positive correlations with social development; (2) gross motor skills demonstrate the strongest associations (weak-to-strong correlations); and (3) fine motor skills exhibit more modest (weak-to-moderate) relationships. The evidence highlights significant disparities in research attention across social development domains. Self-perception has received disproportionate investigation, followed by peer relationships, problem behaviors, negative emotions, and adaptive behaviors, with emotional recognition remaining markedly understudied. These gaps and methodological limitations point to clear directions for future work, as discussed above. For children exhibiting social development challenges, targeted motor skill interventions, particularly those emphasizing gross motor skills, may serve as effective promotive strategies.
